# Serum PTH Associated with Malnutrition Determined by Bioelectrical Impedance Technology in Chronic Kidney Disease Patients

**DOI:** 10.1155/2022/1222480

**Published:** 2022-05-04

**Authors:** Lilin Liu, Lulu Wang, Xiao Wang, Mingxia Xiong, Hongdi Cao, Lei Jiang, Junwei Yang

**Affiliations:** Center for Kidney Disease, Second Affiliated Hospital, Nanjing Medical University, Nanjing, 210003, Jiangsu, China

## Abstract

**Purpose:**

Chronic malnutrition and cachexia are common in chronic kidney disease (CKD), and importance should be given to these complications because they affect the patient's quality of life and prognosis. This study analyzed the correlation between the serum PTH level, nutritional status, and body composition of patients with CKD.

**Methods:**

CKD patients were enrolled in Center for Kidney Disease, Second Affiliated Hospital of Nanjing Medical University, from December 1, 2016, to November 30, 2020. Bioelectrical impedance technology was applied to estimate the body composition. The characteristics of the body composition were compared among different stages of CKD patients, and then the correlation between PTH and body composition was analyzed.

**Results:**

205 CKD patients were enrolled. Twenty-five patients were in stage 1 or 2 of CKD, 78 patients were in stage 3 or 4, 31 patients were in stage 5 without dialysis (referred to as CKD stage 5A), and 71 patients were in stage 5 with dialysis (referred to as CKD stage 5B). Body composition analysis showed that the patients had a phase angle (PA) of 5.02 ± 1.07°, a percentage of body fat (PBF) of 27.74 ± 8.8%, and a skeletal muscle mass index (SMI) of 7.4 ± 1.34 kg/m^2^. PBF peaked in the CKD stage 3/4 group and gradually decreased with the progression of CKD. The PA and SMI differed significantly between the CKD stage 1/2 and stage 5B groups. The proportion of low SMI did not differ significantly between the CKD stage 1/2 and stage 3/4 groups, but it was obviously higher in the CKD stage 5A and 5B groups. PTH was significantly correlated with BMI, hemoglobin, albumin, total cholesterol, triglycerides, and SMI. Binary logistic regression of low SMI showed that the odds ratio for PTH levels was greater than the upper limit of the normal range, which was 11.769 (*p*=0.043, 95% confidence interval: 1.078–128.536), and the model predictive power was 0.986 after correction for age, sex, height, weight, hemoglobin, serum calcium, serum phosphorus, serum total cholesterol, serum triglyceride, and basal metabolic rate.

**Conclusions:**

Bioelectrical impedance analysis might be useful in estimating the nutritional status of CKD patients in terms of fat and muscle parameters. High levels of PTH are an independent risk factor for developing low SMI in CKD patients.

## 1. Introduction

Chronic kidney disease (CKD) is a global health problem, and its prevalence is increasing every year due to the change in the disease spectrum. Chronic malnutrition and cachexia are common in CKD patients, and importance should be given to these complications because they affect the patient's quality of life and may even be associated with depression [1]. Severe protein malnutrition significantly increases the mortality rate of CKD patients [2]. In contrast to malnutrition due to other causes, CKD patients often develop an imbalance between energy requirements and nutritional intake, leading to loss of protein and fat mass, which cannot be readily corrected by dietary supplementation regimens [3].

Previous physiological studies [4, 5] have shown that parathyroid hormone (PTH)/PTH-related protein (PTHrP) induces browning of white fat, increases the basal metabolic rate, affects muscle mass, and have verified the upregulation of thermogenic gene transcription in patients with primary hyperparathyroidism. CKD patients also have high levels of circulating PTH. However, the relationship between PTH and the nutritional status and body composition of CKD patients remains unknown. Traditional nutritional indicators (e.g., albumin, prealbumin, and cholesterol) are generally used to assess the overall nutritional status, while body composition analyzers can provide a wealth of information regarding the basal metabolic rate and the mass and distribution of muscle and fat through bioelectrical impedance technology [6].

Therefore, the purpose of this study was to analyze the nutritional status and body composition of CKD patients using bioelectrical impedance technology, investigate the differences in the nutritional status among patients at different stages of CKD, and analyze the correlation between PTH levels and body composition of CKD patients.

## 2. Materials and Methods

### 2.1. Study Population and Setting

The participants were enrolled from hospitalized patients in Center for Kidney Disease, Second Affiliated Hospital of Nanjing Medical University, from December 1, 2016, to November 30, 2020. The inclusion criteria included participants aged above 18 years old and diagnosed with CKD according to KDIGO guidelines [7]. The exclusion criteria included: (1) acute infection, (2) life expectancy of less than 1 year due to decompensated liver cirrhosis or malignant tumor, and (3) malignant hypertension (SBP ≥ 180 mmHg or DBP ≥ 110 mmHg with clinical symptoms). All participants signed the informed consent, and this study was certified and approved by the Ethics Committee of the Second Affiliated Hospital of Nanjing Medical University (Document Number: [2015] KY052).

### 2.2. Bioelectrical Impedance Analysis of Body Composition

After seated for at least 20 minutes, the participants were examined for human body composition using the biological resistance technology (InBody S10, Korea). Water drinking was forbidden for 2 hours before the examination. Hemodialysis patients were examined half an hour after hemodialysis. Based on the nutritional content estimated by InBody, the bioelectrical impedance results were categorized into three types of nutritional indicators. Total nutritional indicators included the following: phase angle (PA), body cell mass (BCM), protein, and basal metabolic rate (BMR); fat indicators included percent body fat (PBF), visceral fat area (VFA), and body fat mass (BFM); and muscle indicators included fat free mass (FFM), soft lean mass (SLM), skeletal muscle mass (SMM), and skeletal muscle index (SMI) [8, 9]. Here, low PA was defined as less than 5 in men and less than 4.6 in women [10], and low SMI was defined as less than 7 kg/m^2^ in men and less than 5.7 kg/m^2^ in women [11].

### 2.3. General Materials and Laboratory Tests

The demographic and medical information of all subjects was collected. Fasting blood was sampled on the morning of the same day for routine laboratory examination, including hemoglobin, serum albumin, serum total cholesterol, serum triglyceride, serum urea nitrogen, serum creatinine, serum phosphorus, serum calcium, and plasma PTH. PTH was detected by chemiluminescence immunoassay (Beckman Dx1 800 Immunoassay System, America), and the upper limit of the normal detection range, 88 mmol/L, was used as the cut-off value.

### 2.4. Statistical Analyses

The data of normal distribution were described in the form of mean ± standard deviation, the data of non-normal distribution were described in the form of median (interquartile range), and the classified variables were described in the form of frequency (percentage). *T*-test was used to compare the continuous variables of normal distribution between two groups, one-way ANOVA and LSD post hoc tests were used to compare among three or more groups, and nonparametric and Bonferroni's post hoc tests were used to compare the continuous variables of non-normal distribution. The chi-square test was used to compare the rates. The correlation between variables was analyzed by Pearson correlation analysis, in which the non-normal distribution data were transformed into normal distribution data by natural logarithm. Binary logistic regression was used to explore the risk factors of low SMI. All analyses were performed with SPSS 26.0. It is statistically significant when the *p* value is <0.05.

## 3. Results

### 3.1. Basic Information

This study enrolled 205 CKD patients, who were divided into groups based on their CKD stage. Twenty-five patients were in stage 1 or 2 CKD, 78 were in stage 3 or 4, 31 were in stage 5 without dialysis (referred to as CKD stage 5A), and 71 were in stage 5 with dialysis (referred to as CKD stage 5B). Sixty-two percent of the patients were males with a median age of 56 years old. Diabetic nephropathy was the most common etiology of CKD (30.7%) among the patients, followed by glomerulonephropathy (24.9%). As shown in Table 1, there were statistical differences in age, body mass index (BMI), and etiology among groups, but there were no statistical differences in sex ratio.

### 3.2. Differences in Nutritional Indicators among Patients in Different CKD Stages

As summarized in Table 1, CKD patients had a mean BMI of 24.93 kg/m^2^, a median serum albumin level of 39.7 g/L, a median serum total cholesterol level of 4.14 mmol/L, and a median serum triglyceride level of 1.69 mmol/L. As summarized in Table 2, body composition analysis showed that the patients had a PA of 5.02 ± 1.07°, a PBF of 27.74 ± 8.8%, and a/an SMI of 7.4 ± 1.34 kg/m^2^. There were significant differences in the levels of overall nutritional and muscle indicators among the four groups, except for serum albumin level and proportion of patients with low PA. Apart from BFM, there were no significant differences in body fat indicators among the groups.

**Figure 1:**
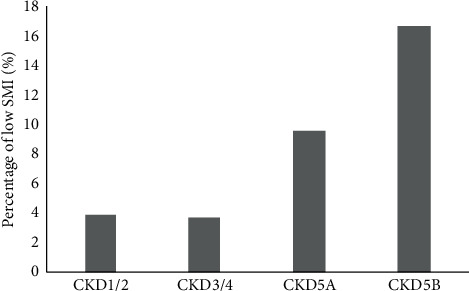
Percentage of low SMI at different CKD stage groups. (*p*value = 0.037).

With the progression of CKD, BMI, total cholesterol, and triglycerides decreased gradually. The albumin level varied greatly among the patients in the CKD stage 1/2 group, but the level increased in the other three groups with the progression of CKD. A significant difference was present only between the groups of CKD stage 3/4 and stage 5B. PBF peaked in the patients of CKD stage 3/4 and gradually decreased with the progression of CKD. PA and SMI differed significantly between the groups of CKD stages 1/2 and 5B, but not between the groups of CKD stages 3/4 and 5A. Further analysis (Figure 1) revealed that the proportion of low SMI did not differ significantly between the groups of CKD stages 1/2 and 3/4, but it was obviously higher in the groups of CKD stages 5A and 5B.

### 3.3. Correlation Analysis of PTH and Nutritional Indicators

The mean PTH level of the patients was 103.5 pg/ml at the time of enrollment, and it showed a marked increase with the progression of CKD. PTH was significantly correlated with BMI, hemoglobin, albumin, total cholesterol, triglycerides, and SMI but not with PA, PBF, and BMR (Table 3).

### 3.4. Risk Factors for Low SMI

The low-SMI group was compared with the normal SMI group (Table 4). Binary logistic regression of low SMI (Table 5) showed that the odds ratio for PTH levels greater than the upper limit (88 pg/ml) of the normal range was 11.769 (*p* = 0.043, 95% confidence interval: 1.078–128.536), and the model predictive power was 0.986 (Table 5) after correction for age, sex, height, weight, hemoglobin, serum calcium, serum phosphorus, serum total cholesterol, serum triglyceride, and basal metabolic rate.

## 4. Discussion

The nutritional status of the patients gradually declined with the progression of CKD, and the changes in the body composition were manifested mainly as the decrease in SMI, while the changes in body fat indicators were not considerable. The proportion of hemodialysis patients with low PA and SMI was significantly higher than that of nondialysis patients at any CKD stage. High levels of PTH were an independent risk factor for developing low SMI in CKD patients.

CKD patients often develop a unique nutritional imbalance, resulting from increase in energy requirements (caused by catabolism and chronic inflammation) with paradoxical reduction in appetite, for which the International Society of Renal Nutrition and Metabolism proposed the concept of protein-energy wasting (PEW) in 2008 [12]. PEW is among the strongest predictors of mortality in CKD patients (hazard ratio 3.03) [13]. Diagnosis and treatment of PEW is a key step in the clinical, nutritional, and overall management of CKD. At present, the diagnosis of PEW is established on the basis of changes in serum biochemical indices, BMI, muscle mass, and dietary intake [12]. Traditional methods of somatometry, such as upper arm circumference measurement and skinfold measurements, for evaluating muscle mass and judging changes in food intake lack uniformity, interrater reliability, and accuracy, while dynamic changes in the water load of hemodialysis patients greatly impact the body weight. On the other hand, assessment of skeletal muscle mass for all CKD patients through radionuclide imaging [11] has potential health and safety risks due to its radioactivity and results in an excessive economic burden on the healthcare system, which limits its wide and flexible application. Bioelectrical impedance technology was adopted in this study to assess the characteristics and changes in the body composition of CKD patients. This technology is convenient, noninvasive, safe, repeatable, and cost-efficient. It can be effectively employed in lieu of the dual-energy X-ray method [14] to estimate objectively and reliably the amount of water, muscle, and fat mass present in the bodies of CKD patients, as well as metabolic carts [15] to estimate their basal metabolic rate, which reflects the nutritional status of these patients, thereby having an increased clinical value for regular dynamic assessment in CKD patients.

A high PTH level is an important risk factor for poor prognosis in CKD patients. During the development and progression of CKD, PTH not only causes bone demineralization but also affects the proper functioning of the brain, heart, and immune system [16]. This clinical analysis revealed that PTH was associated with the nutritional status, especially the muscle mass of CKD patients. The proportion of patients with low SMI was high in the group of CKD stage 5B, while further analysis revealed relatively normal or even slightly high levels of hemoglobin, albumin, and serum phosphorus in these patients, thereby indicating adequate nutritional intake. However, the prevalence of sarcopenia is significantly higher in patients undergoing hemodialysis due to accelerated protein catabolism despite adequate nutrition [17]. Good muscle function is one of the key factors that aids CKD patients in resuming their daily activities. Considering that dietary interventions alone may not improve the nutritional status and muscle function of hemodialysis patients, it is necessary to diagnose malnutrition and PEW early, so that appropriate treatment can be provided. The effect of PTH on muscle tissue is not specific to CKD patients, and similar phenomena have been observed in the experimental models of tumors [4, 5] in patients with primary hyperparathyroidism [4], in female patients with vitamin *D* deficiency [18], and in the elderly population [19], while this association has not been observed among the general population [20]. From a physiological perspective, PTH/PTHrP activates protein kinase A and thermogenic genes such as uncoupling protein 1 (Ucp1), induces browning of white adipocytes, increases basal metabolic rate, and mediates energy wasting, which in turn leads to muscular atrophy and a decline in muscle function [4, 21]. At the cellular level, this manifests as a decrease in both mitochondrial activity and high-energy phosphate content in muscle tissue [22]. The present study provides clinical evidence regarding the effect of high PTH levels on muscle tissue, which may direct future research into the treatment of sarcopenia in CKD patients.

In the present study, albumin levels first increased and then decreased with the progression of CKD, which was different from the trend for most nutritional indicators. The discrepancy may be attributed to the fact that the underlying conditions leading to CKD mainly include diabetic nephropathy and primary glomerulonephritis, which frequently cause proteinuria. The degree of proteinuria correlates with the patient's renal function outcomes [23], which explains the high degree of proteinuria in CKD patients before dialysis. In contrast, as residual renal function is lost when dialysis is started, urine output gradually decreases and the patient's dietary restrictions become fewer than before, allowing for the rise and maintenance of serum albumin levels. Therefore, CKD stage 3/4 patients exhibited the lowest mean level of serum albumin in this study.

There are some limitations in this study. First of all, this was a cross-sectional study and, thus, unable to reveal causality. Further research is needed to confirm whether intervention to reduce PTH levels can serve as an effective treatment for malnutrition and PEW in CKD patients. In view of the U-shaped relationship curve between the PTH level and the mortality risk of CKD patients [24], it is not clear whether simple parathyroidectomy will be able to improve the short- and long-term prognosis of patients. Moreover, the optimal target range for PTH level that should be achieved by using calcimimetics and activated vitamin *D* in order to reduce the risk of developing low SMI is not known. These issues need to be resolved through long-term follow-up. Secondly, BIA is an indirect assessment method, and it can estimate but not measure body composition. In previous research, BIA was proven to apply well in normal hydration CKD patients, which was as reliable as DXA [25]. Meanwhile, CKD patients may have a higher BCM/FFM ratio than the normal population [26]. In a similar research of hemodialysis patients, BCM was 28.0 ± 6.1 kg, FFM was 43.8 ± 9.1 kg, and the BCM/FFM ratio was around 65% [27]. We think it may be related to the changes in fluid volume and cell membrane stability of CKD patients. Hence, we controlled the possible overhydration factors to assure accuracy. Thirdly, unlike previous physiological research, this study did not observe a correlation of PTH levels with body fat indicators (except for serum total cholesterol) and basal metabolic rate. However, this finding does not necessarily confirm the absence of such a correlation. For ethical reasons, it is not possible to obtain adipose tissue from deeper sites from patients to determine whether the expression of thermogenic genes is upregulated. Therefore, the lack of observable correlation may be interpreted as arising from the possible transient effects of PTH on fat and basal metabolic rate as well as the relatively small sample size in this study. A larger sample size is needed to further verify the above observations and postulation. In addition, the self-controlled comparison of patients after parathyroidectomy may provide some evidence for the effects of PTH on fats and basal metabolic rate. Therefore, subsequent clinical research should focus on the establishment of a reasonable control range for PTH and the observation of post-parathyroidectomy changes in patients' body composition.

## 5. Disclosure

A preprint of this article has been published previously (Lilin Liu et al., 2021 [28])

## 6. Conclusions

In summary, bioelectrical impedance might usefully estimate the nutritional status of CKD patients in terms of fat and muscle parameters. High levels of PTH are an independent risk factor for developing low SMI in CKD patients. It is necessary to pay more attention to the nutritional management of CKD patients and the remodeling of fat and muscle distribution. Moreover, PTH may be a potential therapeutic target for treating sarcopenia in CKD patients.

## Figures and Tables

**Table 1:** Basic indicators of different CKD stage groups.

	Total, *n* = 205	CKD1/2, *n* = 25	CKD3/4, *n* = 78	CKD5A, *n* = 31	CKD5B, *n* = 71	*p* value
Age, yr, median (IQR)	56 (46, 67)	56 (39.5, 63)	62 (47.75, 68)	58 (46, 67)	53 (43, 64) &	0.036
Male, *n* (%)	127 (62%)	20 (80%)	51 (65.4%)	14 (45.2%)	42 (59.2%)	0.051
Body mass index, kg/m2, mean ± SD	24.93 ± 4.1	25.9 ± 4.53	25.82 ± 3.57	25.14 ± 3.17	23.52 ± 4.51 !,$	0.003
CKD etiology, *n* (%)						<0.001
Diabetic nephropathy	63 (30.7%)	9 (36%)	32 (41%)	13 (41.9%)	9 (12.7%)	
Hypertensive nephropathy	28 (13.7%)	5 (20%)	11 (14.1%)	4 (12.9%)	8 (11.3%)	
Glomerulonephropathy	51 (24.9%)	7 (28%)	24 (30.8%)	7 (22.9%)	13 (18.3%)	
Others	22 (10.7%)	4 (16%)	7 (9%)	4 (12.9%)	7 (9.9%)	
Unknown	41 (20%)	0 (0%)	4 (5.1%)	3 (9.7%)	34 (47.9%)	
Parathyroid hormone, pg/ml, median (IQR)	103.5 (49.05, 452.25)	45.3 (25, 61.3)	57.25 (39.88, 94.85)	216.3 (100.5, 351.2)#,¶	773.1 (216.6, 1750.7) !,$	<0.001
Hemoglobin, g/L, mean ± SD	109.58 ± 23.06	125.12 ± 20.06	115.26 ± 21.63^*∗*^	93.03 ± 15.64#,¶	105.08 ± 23 !,$,&	<0.001
Albumin, g/L, median (IQR)	39.7 (34.95, 43.5)	40.2 (35.5, 45.5)	38.75 (32.95, 42.48)	38.9 (34.8, 43)	41.5 (37.9, 44)	0.056
Total cholesterol, mmol/L, median (IQR)	4.14 (3.42, 4.85)	4.74 (3.86, 5.41)	4.37 (3.75, 5.05)	4.21 (3.49, 4.83)	3.62 (3.11, 4.29) !,$	<0.001
Triglyceride, mmol/L, median (IQR)	1.69 (1.16, 2.44)	1.95 (1.26, 2.85)	1.82 (1.32, 2.82)	1.69 (1.16, 2.44)	1.49 (0.92, 2.08) $	0.036
Phosphorus, mmol/L, median (IQR)	1.35 (1.11, 1.88)	1.1 (0.95, 1.18)	1.18 (1.04, 1.36)	1.43 (1.3, 1.59)#,¶	2.11 (1.59, 2.44) !,$,&	<0.001
Calcium, mmol/L, median (IQR)	2.19 (2.09, 2.36)	2.16 (2.08, 2.26)	2.17 (2.09, 2.29)	2.12 (2, 2.17)	2.34 (2.16, 2.55) !,$,&	<0.001

IQR, interquartile range; ^*∗*^=*p* < 0.05 between CKD1/2 and CKD3/4; ^#^=*p* < 0.05 between CKD1/2 and CKD5A; ^#^=*p* < 0.05 between CKD1/2 and CKD5A; ^¶^=*p* < 0.05 between CKD3/4 and CKD5A; ^!^=*p* < 0.05 between CKD1/2 and CKD5B; ^$^=*p* < 0.05 between CKD3/4 and CKD5B; ^&^=*p* < 0.05 between CKD5A and CKD5B.

**Table 2:** Nutritional indicators of different CKD stage groups.

	Total, *n* = 205	CKD1/2, *n* = 25	CKD3/4, *n* = 78	CKD5A, *n*= 31	CKD5B, *n* = 71	*p* value
**Total Nutritional Indicators**						
weight, kg	68.6 (58.1, 77.95)	73.1 (58.1, 83.75)	72.8 (61.95, 79.13)	68.5 (58, 78.1)	63.9 (53.3, 70.8)!,$	0.001
PA, °, mean ± SD	5.02 ± 1.07	5.37 ±1.22	5.14 ± 1.05	5.15 ± 0.88	4.72 ± 1.07!,$	0.02
Low PA, n (%)	70 (34.1%)	5 (20%)	23 (29.5%)	10 (32.3%)	32 (45.1%)	0.079
Body cell mass, kg, median (IQR)	31.9 (26.2, 35.6)	34.4 (29.4, 40.05)	32.7 (26.78, 36.75)	30.6 (25.6, 37)	29.6 (25.8, 33.9)!	0.025
Protein, kg, median (IQR)	9.6 (7.9, 10.8)	10.4 (8.85, 12.1)	9.9 (8.1, 11.1)	9.2 (7.7, 11.2)	9 (7.7, 10.2)!	0.024
Basal metabolic rate, kcal, median (IQR)	1429 (1255.5, 1563)	1502 (1338, 1685)	1458.5 (1269.75, 1577.75)	1378 (1215, 1604)	1355 (1235, 1511)	0.038
**Fat Indicators**						
Percent body fat, %, mean ± SD	27.74 ± 8.8	26.86 ± 10.48	29.35 ± .84	28.46 ± 8.65	25.97 ± 9.03	0.113
Visceral fat area, cm^2^, median (IQR)	84.2 (63.85, 114.45)	101.2 (50.95, 133.85)	86.5 (73.95, 125.6)	83.9 (56.4, 113.5)	78.2 (55.1, 107.8)	0.235
Body fat mass, kg, mean ± SD	19.38 ± 8.2	20.57 ± 10.48	20.94 ± 7.14	19.42 ± 6.75	17.23 ± 8.66$	0.04
**Muscle Indicators**						
Fat free mass, kg, median (IQR)	49 (40.95, 55.25)	52.4 (44.8, 60.9)	50.4 (41.68, 55.9)	46.6 (39.1, 57.1)	45.6 (40, 52.8)	0.037
Soft lean mass, kg, median (IQR)	46.2 (38.5, 52.05)	49.8 (42.45, 57.35)	47.6 (39.08, 52.95)	44 (36.9, 53.9)	43.1 (37.7, 50.1)	0.04
Skeletal muscle mass, kg, median (IQR)	27 (21.9, 30.4)	29.3 (24.75, 34.45)	27.8 (22.38, 31.45)	25.9 (21.3, 31.7)	24.9 (21.5, 28.9)!	0.024

IQR, interquartile range; PA, phase angle; ^!^=*p* < 0.05 between CKD1/2 and CKD5B; ^$^=*p* < 0.05 between CKD3/4 and CKD5B.

**Table 3:** Correlation between ln PTH and nutritional indicators.

lnPTH		
Variables	r (95%CI)	*p* value
BMI	−0.141 (−0.274, −0.002)	0.043
Hb	−0.139 (−0.267, −0.005)	0.046
lnAlb	0.181 (0.061, 0.299)	0.009
lnTc	−0.221 (−0.332, −0.102)	0.001
lnTg	−0.190 (−0.316, −0.056)	0.006
PA	−0.086 (−0.221, 0.049)	0.220
lnBMR	−0.110 (−0.237, 0.012)	0.117
PBF	−0.113 (−0.240, 0.022)	0.105
SMI	−0.141 (−0.264, −0.020)	0.043

PTH,parathyroid hormone; Hb, hemoglobin; Alb, albumin; Tc, total cholesterol; Tg, triglyceride; PA, phase angle; BMR, basal metabolic rate; PBF, percent body fat; SMI, skeletal muscle index.

**Table 4:** Comparison between low and normal SMI groups.

Variables	Low SMI, *n* = 19	Normal SMI, *n* = 186	*P* Value
**Basic Indicators**			
Age, yr, median (IQR)	56 (34, 71)	56.5 (46, 67)	0.636
Male, *n* (%)	3 (15.8%)	124 (66.7%)	<0.001
Body mass index, kg/m^2^, mean ± SD	20.17 ± 3.24	25.42 ± 3.86	<0.001
CKD etiology, *n* (%)			0.080
Diabetic nephropathy	3 (15.8%)	60 (32.3%)	
Hypertensive nephropathy	1 (5.3%)	27 (14.5%)	
Glomerulonephropathy	4 (21.1%)	47 (25.3%)	
Others	3 (15.8%)	19 (10.2%)	
Unknown	8 (42.1%)	33 (17.7%)	
CKD stage, *n* (%)			0.037
1 + 2	1 (5.3%）	24 (12.9%)	
3 + 4	3 (15.8%)	75 (40.3%)	
5A	3 (15.8%)	28 (15.1%)	
5B	12 (63.2%)	59 (31.7%)	
Parathyroid hormone, pg/ml, median (IQR)	250.4 (110.4, 773.1)	95.45 (47.35, 441.03)	0.056
Parathyroid hormone>88mmol/L	16 (84.2%)	104 (55.9%)	0.017
Hemoglobin, g/L, mean ± SD	107 ± 18.72	109.84 ± 23.49	0.611
Albumin, g/L, median (IQR)	39.7 (34.8, 44.9)	39.7 (35.03, 43.5)	0.836
Total cholesterol, mmol/L, median (IQR)	4.26 (3.55, 5.14)	4.14 (3.35, 4.84)	0.418
Triglyceride, mmol/L, median (IQR)	1.46 (0.99, 2.22)	1.71 (1.17, 2.44)	0.626
Phosphorus, mmol/L, median (IQR)	1.28 (1.08, 1.94)	1.36 (1.12, 1.86)	0.863
Calcium, mmol/L, median (IQR)	2.19 (2.12, 2.55)	2.18 (2.07, 2.35)	0.326
**Total Nutrition Indicators**			
PA, °, median (IQR)	4.2 (3.4, 4.7)	5.2 (4.38, 5.9)	<0.001
Low PA, *n* (%)	14 (73.7%)	56 (30.1%)	<0.001
Body cell mass, kg, mean ± SD	22.43 ± 1.76	32.76 ± 6.61	<0.001
Protein, kg, median (IQR)	6.9 (6.3, 7.1)	9.85 (8.2, 10.95)	<0.001
Basal metabolic rate, kcal, median (IQR)	1134 (1086, 1177)	1463 (1285.75, 1575.75)	<0.001
**Fat Indicators**			
Percent body fat, %, mean ± SD	28.37 ± 10.12	27.68 ± 8.68	0.745
Visceral fat area, cm^2^, median (IQR)	68.7 (49, 122.1)	84.5 (67.7, 114.18)	0.105
Body fat mass, kg, mean ± SD	14.93 ± 7.69	19.83 ± 8.14	0.013
**Muscle Indicators**			
Fat free mass, kg, median (IQR)	35.4 (33.2, 37.4)	50.6 (42.4, 55.83)	<0.001
Soft lean mass, kg, median (IQR)	33.1 (31.2, 35)	47.85 (39.98, 52.65)	<0.001
Skeletal muscle mass, kg, median (IQR)	18.6 (17.1, 19.6)	27.6 (22.8, 31.18)	<0.001
SMI, kg/m^2^, median (IQR)	5.4 (5, 5.6)	7.6 (6.7, 8.3)	<0.001

IQR, interquartile range; PA, phase angle; SMI, skeletal muscle mass.

**Table 5:** Relationship between PTH and low SMI (Binary logistic regression analysis).

	Model 1			Model 2		
	OR (95%CI)	*p*	C statistic	OR (95%CI)	*p*	C statistic

PTH ≤ 88pg/ml	0.238 (0.067-0.844)	0.026	0.641	0.085 (0.008-0.928)	0.043	0.986
PTH＞88pg/ml	4.205 (1.185-14.924)			11.769 (1.078-128.536)		

## Data Availability

The data that support the findings of this study are available from the corresponding author upon reasonable request.

## Abbreviations

CKD: Chronic kidney disease
IQR: Interquartile range
PTH: Parathyroid hormone
Hb: Hemoglobin
Alb: Albumin
Tc: Total cholesterol
Tg: Triglyceride
PA: Phase angle
BMR: Basal metabolic rate
PBF: Percent body fat
SMI: Skeletal Muscle Index.

## Supplementary Materials

Supplementary Materials STable 1: basic indicators of different genders. STable 2: nutritional indicators of different genders.
