# Supplementary material for: Serum PTH Associated with Malnutrition Determined by Bioelectrical Impedance Technology in Chronic Kidney Disease Patients
Source: Int J Endocrinol. 2022 May 4;2022:1222480. doi: 10.1155/2022/1222480 (PMC9095397; doi:10.1155/2022/1222480)
Supplement: Supplementary Materials — STable 1: basic indicators of different genders. STable 2: nutritional indicators of different genders. [file 1222480.f1.pdf]

STable 1. Basic indicators of different genders

	Total, n=205	Female, n=78	Male, n=127	P value
Age, yr, median (IQR)	56 (46, 67)	59.5(44, 67.25)	56(47, 65)	0.36
Body mass index, kg/m^2, mean±SD	24.93±4.1	24.09±3.98	25.45±4.1	0.021
CKD etiology, n (%)				0.002
Diabetic nephropathy	63 (30.7%)	22(28.2%)	41(32.3%)	
Hypertensive nephropathy	28 (13.7%)	3(3.8%)	25(19.7%)	
Glomerulonephropathy	51 (24.9%)	29(37.2%)	22(17.3%)	
Others	22 (10.7%)	8(10.3%)	14(11%)	
Unknown	41 (20%)	16(20.5%)	25(19.7%)	
Parathyroid hormone , pg/ml, median (IQR)	103.5 (49.05, 452.25)	137.75(68.95, 450.1)	95.3(45, 467.3)	0.36
Hemoglobin, g/L, mean±SD	109.58±23.06	100.94±20.53	114.88±23	<0.001
Albumin, g/L, median (IQR)	39.7 (34.95, 43.5)	38.65(33.4, 42.7)	40.2(35.8, 44.1)	0.084
Total cholesterol, mmol/L, median (IQR)	4.14 (3.42, 4.85)	4.36(3.72, 5.06)	3.92(3.19, 4.74)	0.002
Triglyceride, mmol/L, median (IQR)	1.69 (1.16, 2.44)	1.81(1.27, 2.4)	1.62(1.11, 2.51)	0.385
Phosphorus, mmol/L, median (IQR)	1.35 (1.11, 1.88)	1.4(1.17, 1.66)	1.32(1.06, 1.97)	0.51
Calcium, mmol/L, median (IQR)	2.19 (2.09, 2.36)	2.17(2.11, 2.36)	2.2(2.07, 2.37)	0.728

IQR, interquartile range.

STable 2. Nutritional indicators of different genders

	Total, n=205	Female, n=78	Male, n=127	P value
**Total Nutritional Indicators**				
weight, kg	68.6(58.1, 77.95)	58.9(51.15, 67.5)	73.5(64.5, 81.4)	<0.001
PA, °, mean±SD	5.02±1.07	4.67±0.96	5.24±1.09	<0.001
Low PA, n (%)	70 (34.1%)	37(47.4%)	33(26%)	0.002
Body cell mass, kg, median (IQR)	31.9 (26.2, 35.6)	25.55(23.65, 27.2)	34.6(32.1, 39.1)	<0.001
Protein, kg, median (IQR)	9.6 (7.9, 10.8)	7.7(7.1, 8.2)	10.5(9.7, 11.8)	<0.001
Basal metabolic rate, kcal, median (IQR)	1429 (1255.5, 1563)	1228.5(1164, 1277)	1523(1437, 1670)	<0.001
**Fat Indicators**				
Percent body fat, %, mean±SD	27.74±8.8	32.32±8.4	24.93±7.82	<0.001
Visceral fat area, cm^2, median (IQR)	84.2 (63.85, 114.45)	99.4(65.78, 128.35)	82.2(63.6, 103.3)	0.025
Body fat mass, kg, mean±SD	19.38±8.2	20.1±7.79	18.94±8.45	0.329
**Muscle Indicators**				
Fat free mass, kg, median (IQR)	49 (40.95, 55.25)	39.75(36.8, 41.95)	53.4(49.4, 60.2)	<0.001
Soft lean mass, kg, median (IQR)	46.2 (38.5, 52.05)	37.3(34.6, 39.68)	50.4(46.7, 56.9)	<0.001
Skeletal muscle mass, kg, median (IQR)	27 (21.9, 30.4)	21.25(19.48, 22.8)	29.5(27.2, 33.6)	<0.001

IQR, interquartile range; PA, phase angle.
